# Code of Ethics

**Published:** 1881-06

**Authors:** 

**Affiliations:** Baltimore


Code of Ethics.—A well-bred gentleman, appreciative of his own rights and obligations to society, will experience no difficulty in yielding obedience to the demands and requirements of conventional life in any of its departments, and will be in no need of a written code or formulated laws to enable him to comprehend the extent of his privileges and duties as they impinge or overlap the rights and privileges of his neighbors engaged in the same or similar pursuit or avocation with himself. He will intuitively perceive where his rights and privileges cease and those of his neighbors begin, and govern himself accordingly, yielding to them all that he requires from others in return, with no thought of the demands or protection of any written code or laws.

But, unfortunately, the harmony of social life and professional intercourse are often interrupted, and discord produced, by those who have entered one or both in some other and less carefully organized way than that which gentility requires, and are consequently incapable of appreciating the unwritten code which governs the conduct of the well-bred gentleman.

As, however, the former class exists, and its representatives are to be found in the various professions and avocations of life, a written code for *their* government becomes necessary and important to the well-being of all who are, or may be, compelled to associate with them. Did such a written code suffice to restrain and keep within its prescribed limits *all* for whom it was intended much good to the whole body embraced within its purview would result to all its members. But, unfortunately, the wolves in sheep's clothing, having once gotten into the medical fold, use their association or membership in a medical society to screen and protect themselves in their nefarious and dirty work. An instance of this character has been brought to our notice, in which a certain party actually begs an editor, from time to time, to notice his exploits in medicine, or to publish under his name the literary work of others—such as poetry, etc. But, as though these were not enough to gratify an overweening desire to see himself and imaginary exploits in print, has actually hired their publication in a newspaper. And yet the party is said to be a member of two or three local societies and of the American Medical Association. The Code of Ethics of some of these several societies ought certainly to have scope enough to reach such characters. There are other members of medical societies who, we are informed, seek to advance *their* professional interests in other ways not recognized by the Code of Ethics. But, as this communication is long enough, we will reserve further remarks for another occasion.

Yours, RACHIS.

BALTIMORE, June, 1881.

